# Pathological complete response in advanced gastric stromal tumor after imatinib mesylate therapy: a case report

**DOI:** 10.1186/1752-1947-5-197

**Published:** 2011-05-21

**Authors:** Omar El Mesbahi, Sami Aziz Brahmi, Yousra Akasbi, Fatima Zahra El Mrabet, Anas Touyar, Abdelmalek Ossaden, Kaoutar Znati, Khalid Maazaz, Affaf Amarti, Siham Tizniti, Khalid Ait Taleb, Adil Ibrahimi

**Affiliations:** 1 Medical Oncology Department, Hassan II University Hospital, Fez, Morocco; 2 Surgery Department, Hassan II University Hospital, Fez, Morocco; 3 Pathology Department, Hassan II University Hospital, Fez, Morocco; 4 Radiology Department, Hassan II University Hospital, Fez, Morocco; 5 Gastroenterology Department, Hassan II University Hospital, Fez, Morocco

## Abstract

**Introduction:**

Gastrointestinal stromal tumors are a rare neoplasm exhibiting, in most cases, mutations of *c-kit*. Imatinib mesylate is the standard treatment for patients who have advanced gastrointestinal stromal tumors. Although the response rate in patients treated with imatinib mesylate in prospective clinical studies is above 50%, a complete response is very rare. We report the case of a patient with a gastric gastrointestinal stromal tumor who had a pathological complete response after neoadjuvant treatment with imatinib mesylate.

**Case presentation:**

We report the case of a 54-year-old Arab woman with a gastrointestinal stromal tumor who had a pathological complete response after neoadjuvant treatment with imatinib mesylate.

**Conclusion:**

The pathological examination of our patient documented a complete pathological response after imatinib therapy. Recently, it has been confirmed that the kinase genotype of *KIT *and *platelet-derived growth factor receptor α *can accurately predict a good response to imatinib mesylate therapy. We propose that this patient had a mutation conferring high sensitivity to imatinib mesylate.

## Introduction

A gastrointestinal stromal tumor (GIST) is a neoplasm exhibiting, in most cases, mutations of *c-kit*. Imatinib mesylate (IM) is the standard treatment for patients who have advanced GISTs, and has recently become the standard treatment in the adjuvant setting as well, but not all patients benefit equally. Although the response rate in patients treated with IM in prospective clinical studies is above 50% [1], complete response is rare. We report a case of a patient with a GIST who had a pathological complete response after neoadjuvant treatment with IM.

### Case presentation

**Figure 1:**
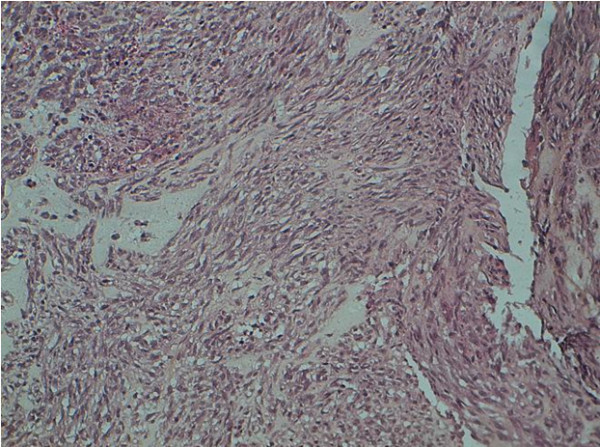
**Histological study revealing spindle cells (original magnification, ×40)**.

**Figure 2:**
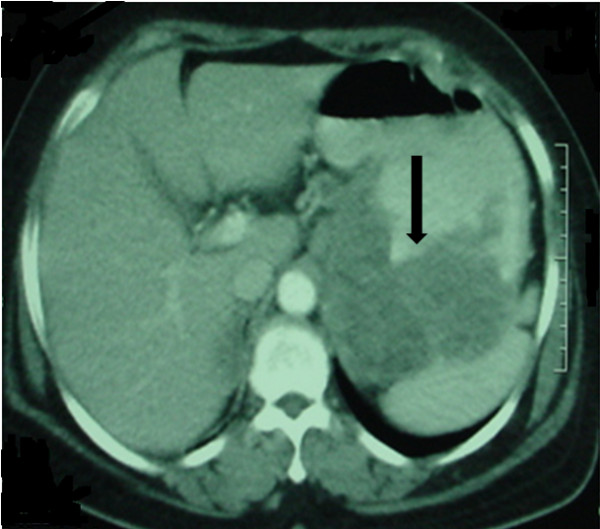
**Computed tomographic scan revealing a large gastric tumor**.

**Figure 3:**
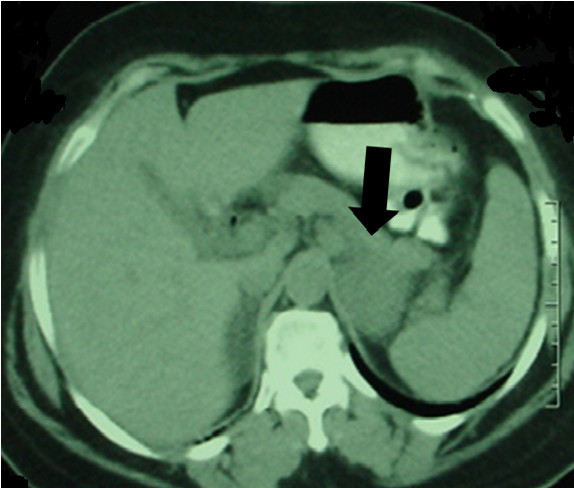
**Computed tomographic scan showing a major radiological response after six months of imatinib treatment**.

A 54-year-old Arab woman presented with a four-month history of epigastralgia and anemia. The endoscopic findings of an examination of the patient's stomach revealed an ulcerative tumor of the fundus, from which the guided biopsy yielded a spindle cell neoplasm with positive immunohistochemical staining using monoclonal antibodies against CD117 (c-kit). The morphologic (Figure 1) and immunohistochemical features of the tumor were consistent with GIST. The molecular biology examination was not done. The abdominal computed tomography scan revealed a heterogeneous mass of the gastric lesser curvature measuring 11 cm × 9 cm × 7.5 cm (Figure 2). The tumor was unresectable; hence IM was indicated at the dose of 400 mg/day. The patient received six months of treatment with IM, which was well tolerated. A radiology-based major response was obtained, thus making the tumor resectable (Figure 3). Fluorodeoxyglucose positron emission tomography (FDG-PET) has proved to be highly sensitive in the early assessment of tumor response. Unfortunately, the patient did not undergo FDG-PET, because it is not available in Morocco. The patient received a partial gastrectomy. The margins of the gastric resection were free of neoplasm.

**Figure 4:**
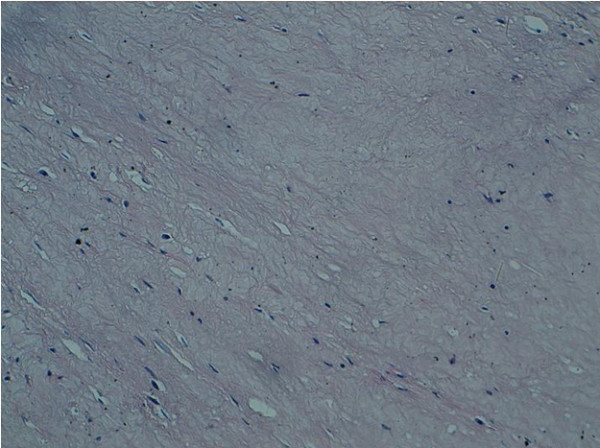
**Total disappearance of tumor cells in the resected specimen after six months of imatinib treatment (original magnification, ×40)**.

No gastric cancer cells were detected in the resected specimen (Figure 4). The patient received one year of IM therapy after the surgery. The patient was in complete remission nine months after the end of IM therapy.

## Discussion

The management of GIST is carried out using a multidisciplinary approach. Standard treatment of localized GIST is complete surgical excision associated (or not) with adjuvant imatinib therapy. If R0 resection is not feasible, or if it can be achieved through less mutilating surgery in the case of cytoreduction, imatinib pretreatment is recommended [2,3]. The benefit of adjuvant imatinib therapy after neoadjuvant therapy is still in exploration. To the best of our knowledge, no studies evaluating this strategy have been reported in the literature. Therefore, our case illustrates complete remission after adjuvant therapy in a patient who received IM as a neoadjuvant treatment. Complete pathological response in resection specimen is rarely reported. After a wide study of the literature, only nine clinical cases have been published of reports of complete pathologic response after imatinib therapy in locally advanced or metastatic GIST [4,5]. Mutational analysis (*KIT/platelet-derived growth factor receptor α (PDGFRA) receptor *mutations) has predictive value for sensitivity to molecular targeted therapy as well as prognostic value, so that its inclusion in the diagnostic work-up of all GISTs is recommended [2]. Patients with exon 11 mutations of *KIT *have been found to have the best response to IM, better than those patients with exon 9 mutations of *KIT *[6]. We suppose that our patient had a mutation which confers a high sensitivity to IM. The mutational analysis in our patient will be done later in our institution's molecular laboratory. The clinical benefit of IM can also be correlated to IM plasma levels in patients with unresectable or metastatic GISTs, as well as a previous study [7] that suggests that a low steady-state plasma level of IM at day 29 after the initiation of IM treatment (<1100 ng/mL) might contribute to drug failure in patients with advanced GISTs. Mutational analysis may help to exclude patients with a less sensitive mutational status (for example, *PDGFRA *D842V mutations) from IM therapy or to tailor a therapy [2] so that it may also help clinicians to make a decision regarding whether to perform surgery or administer neoadjuvant therapy for resectable GISTs.

## Conclusion

The pathological examination of our patient documented a complete response after IM therapy. Recently, it has been confirmed that the kinase genotypes of *KIT *and *PDGFRA α *can accurately predict a good response to IM therapy. We propose that this patient had a mutation conferring high sensitivity to IM.

## Abbreviations

GIST: gastrointestinal stromal tumor; IM: imatinib mesylate; *PDGFRA*: platelet-derived growth factor receptor α.

## Consent

Written informed consent was obtained from the patient for publication of this case report and any accompanying images. A copy of the written consent is available for review by the Editor-in-Chief of this journal.

## Competing interests

The authors declare that they have no competing interests.

## Authors' contributions

EO was the major contributor in writing the manuscript. SAB edited the manuscript and assisted in reviewing the literature. AO and KM were the operating surgeons involved in the case. KZ and AA performed the histological examination. ST performed diagnostic radiology and interpreted the X-rays. AI and AT rendered the initial diagnosis. All authors read and approved the final manuscript.
